# Child, family, and narcissistic political leadership: a comparison of Hitler, Putin, and Trump

**DOI:** 10.3389/fpsyg.2025.1579958

**Published:** 2025-05-21

**Authors:** Yusuf Çifci

**Affiliations:** Department of Political Science and Public Administration, Faculty of Economics and Administrative Sciences, Muş Alparslan University, Muş, Türkiye

**Keywords:** reactive and constructive narcissism, parental and family causes of narcissism, narcissistic political leadership, Hitler, Putin, Trump

## Abstract

**Introduction:**

Every person goes through a period of narcissistic development. Narcissism develops depending on whether children get balanced and adequate care during childhood. Children who get adequate and balanced care develop self-esteem and have constructive narcissistic qualities. Those who do not obtain adequate and balanced care are affected by narcissistic personality disorder and have reactive narcissistic characteristics. Political leaders are no exception to this rule; they were also once children and went through a stage of narcissistic development. This study aims to analyze how Hitler, Putin, and Trump completed their narcissistic developmental stages. In this regard, this study comparatively analyses the causes of these three individuals’ narcissistic political leadership related to their parents, families, and childhood.

**Methods:**

It is not possible to apply psychological tests to political leaders, to sit them on Freud’s couch and psychoanalyse them. However, it is possible to find detailed information on the childhoods and families of political leaders. This study phenomenologically compares the familial reasons for the narcissistic political leadership of Hitler, Putin, and Trump on the basis of the available information. In this study, the phenomenological analysis technique was used based on qualitative information theory.

**Results:**

Hitler, Putin, and Trump grew up in similar families. When these three leaders are compared on the axis of familial causes of narcissism, it is seen that all three leaders were traumatized, experienced frustrations in their childhood that were not appropriate for their age, and grew up with an over-authoritarian father figure in addition to a mother who approached them with compassion. The fact that Hitler and Putin grew up as replacement children and were physically violated by their fathers, whereas Trump was placed in a boarding and disciplined military school at a precisely narcissistic developmental stage, triggered these three leaders to become narcissistic political leaders.

**Discussion:**

It should not be forgotten that Hitler, as a narcissistic political leader, caused the deaths of millions of innocent people. In this sense, it is impossible to evaluate the policies implemented by political leaders and their leadership qualities apart from the family and childhood periods in which they grew up.

## 1 Introduction and methodology

Psychological disorders do not only remain in the individual sphere. Individual psychological disorders often have essential social and political consequences. Especially if this disorder is narcissism and the narcissist is a political leader, the consequences can be even more dire. Narcissism is also a psychological problem with significant social consequences (Twenge and Campbell, 2010). Although it is asserted that narcissism is an urban disease and has become pervasive with capitalism (Layton, 2006, pp. 144–149), this information requires verification. Narcissism, which is named after the myth of Narcissus, has persisted for many years as a disorder of “excessive self-love” (Bushman, 2017). Notably, the correlation between leaders and narcissism has been discussed for a long time. In our days, narcissism is characterized as the personality of the age (Philipson, 1985).

The literature on political leadership and narcissism is voluminous. Because there is a close relationship between leadership and narcissism, and narcissists are very prone to be leaders due to the characteristics caused by narcissism (Deluga, 1997). Many studies have examined the narcissism of political leaders, such as Roman Emperor Suetonius Caligula, Stalin, Saddam Hussein, Genghis Khan, Attila the Hun, Benito Mussolini, and Napoleon (Kakar, 1977; McFarren, 1998; Dörner and Güss, 2011; Bushman, 2017). Because politics unquestionably provides an ideal platform for the expression of narcissism (Hatemi and Fazekas, 2018). It is even claimed that narcissism-induced grandiosity could pave the way for political success (Ahmadian et al., 2017).

History is replete with political events initiated by the personalities of leaders. Nevertheless, the connection between leaders’ personalities and the policies they enforce is often underestimated. Because we classify a human being into (1) a rational person who can act logically and (2) an emotional person who is irrational and acts according to prejudices. Mostly, we prefer to deal with the first person and exclude the influence of the second. We believe that leaders are highly skilled at distinguishing between these two individuals. However, if we consider the evidence of clinical psychology, we know that emotions affect thinking, reasoning, and decision-making processes in every situation. Most of these emotional factors lie rooted in the individual’s past, especially in childhood, and are processed unconsciously (Kakar, 1977, p. 168).

Although we cannot examine human beings, especially leaders, independently of their emotions, the literature assumes that leaders are generally never children or have no problems with their parents. Sitting political leaders on Freud’s couch and listening to them is impossible. Moreover, having political leaders fill out a typical psychological test or questionnaire is impossible. It is tough to obtain a complete psychological profile of political figures due to the lack of direct contact with and access to the respondents (Nai and Maier, 2021). If we take into account the patient registration documents that disappeared after becoming a political leader, as in the case of Hitler (Achtler, 2007), it becomes tough to make a social psychological analysis of political leaders.

Although it is tough to apply psychological tests to political leaders, there is an extensive literature on narcissism and political leadership. Most work on this subject concerns Hitler (Dreijmanis, 2005; Macleod, 2005; Achtler, 2007; Dörner and Güss, 2011; Haque et al., 2012). Hitler is quickly followed by Trump (Aschroft, 2016; Ahmadian et al., 2017; Ott, 2017; Visser et al., 2017; Williams et al., 2020; Hart and Stekler, 2021; Nai and Maier, 2021; Lee, 2023; Maratea, 2024). The least studies on narcissism and leadership are about Putin (Laqueur, 2015; Volkan, 2020). However, this does not show that Putin’s political leadership is less narcissistic, but rather that freedom of expression in Russia is in a precarious situation. As you observe, there are many studies on the narcissistic leadership of Hitler, Putin, and Trump. But none of these studies have centered on the relationship between narcissism, family, and parents. Many of these studies involve quantitative analyses of leaders’ speeches. In this article, I depart slightly from the literature and focus on the extent to which Hitler, Putin, and Trump’s families, family members, and significantly their parents influenced their narcissistic leadership.

Psychiatry’s relationship with politics has often been problematic (Andrade and Campo-Redondo, 2020, p. 1). We could state the same thing about psychology because the power in the hands of psychiatrists is different from that of other scientists. However, the political credentials of psychiatry are not very clean. We know that during the Soviet Union, dissidents were diagnosed with “sluggish schizophrenia,” that in China, dissidents were locked up in a hospital-prison-like place called “Ankang,” and that in the USA, the beating of enslaved people with whips was legitimized by psychiatrists. Experiences during Goldwater’s presidential candidacy in the USA and the subsequent decision of the American Psychiatric Association are also vital in this respect. Because in this decision, it was stated that it was unethical for psychiatrists to express an opinion without examining a person (Andrade and Campo-Redondo, 2020, pp. 3–6).

My study does not violate these ethical rules. Although it is impossible to meet political leaders face to face (Nai and Maier, 2021), this does not change the fact that they were once children and grew up in a family. There is a great deal of evidence about the family, parents, and childhood of many people who are political leaders today or in the past. In this context, in this study, I categorize knowledge about Hitler, Putin, and Trump based on childhood, family, and parents. I make a socio-political critique of leadership on the axis of the family and parental causes of narcissistic political leadership and try to warn about the possible problems that may arise due to leaders’ narcissistic disorders. Considering that similar warnings were made even before Hitler came to power (Kakar, 1977, p. 165), I could argue that this study is crucial.

In this article, I try something that has never been done before in the literature on narcissism and leadership. I am doing a comparative investigation on the familial reasons for the narcissistic leadership of Hitler, Putin, and Trump based on interpretivist epistemology. In this article (1), I use the concepts of healthy and pathological (reactive) narcissism as triangulation points. (2) Based on this triangulation point, I explain the parental and familial causes of unhealthy narcissism one by one. (3) Finally, I make a comparative analysis of the familial and parental reasons for the unhealthy narcissism and narcissistic leadership of Hitler, Putin, and Trump. In the political psychology literature, there are many academic studies analyzing Hitler, Putin, and Trump on the axis of narcissism. However, there is no study in the literature comparing the narcissistic leaderships of these three figures and their familial origins. Therefore, I can claim that this study makes a special contribution to the literature.

## 2 What is reactive and constructive narcissism?

Narcissism is based on a mythological story from Ancient Greece. According to this myth, Narcissus is the son of the river god Kefisos and the water nymph Liriope. Almost everyone who sees Narcissus, who has a rare beauty, falls in love with him. Among those who fall in love with Narcissus is Echo, the daughter of air and earth. Echo, who does not get a response to her love, is left with her voice repeating the last syllable of a word. In retaliation, Nemesis, the goddess of revenge, sends Narcissus to a water source. While quenching his thirst there, Narcissus sees his reflection in the water and falls in love with his own image. Unable to touch his own image and unable to forget his love for himself, Narcissus eventually forgets to eat and drink and falls into the water while trying to hug his own image and drowns (Hirigoyen, 2019). The concept of narcissism is used to describe self-love and is named after Narcissus, who fell in love with his own reflection (Loewenstein, 1997). Narcissus is said to have said, “I burn from the love I feel for myself” (Bushman, 2017).

As can be understood from the story of Narcissus, narcissism is a kind of self-love. The first person to explain the myth of Narcissus by referring to an auto-erotic sexual situation was the British sexologist-physician, Havelock Ellis. According to him, the tendency in narcissus-like cases is that sexual feelings are dissolved and often lost in self-admiration (Ellis, 1898). Narcissists cannot love others; they only want to be reflected by them (McFarren, 1998).

**Figure 1:**
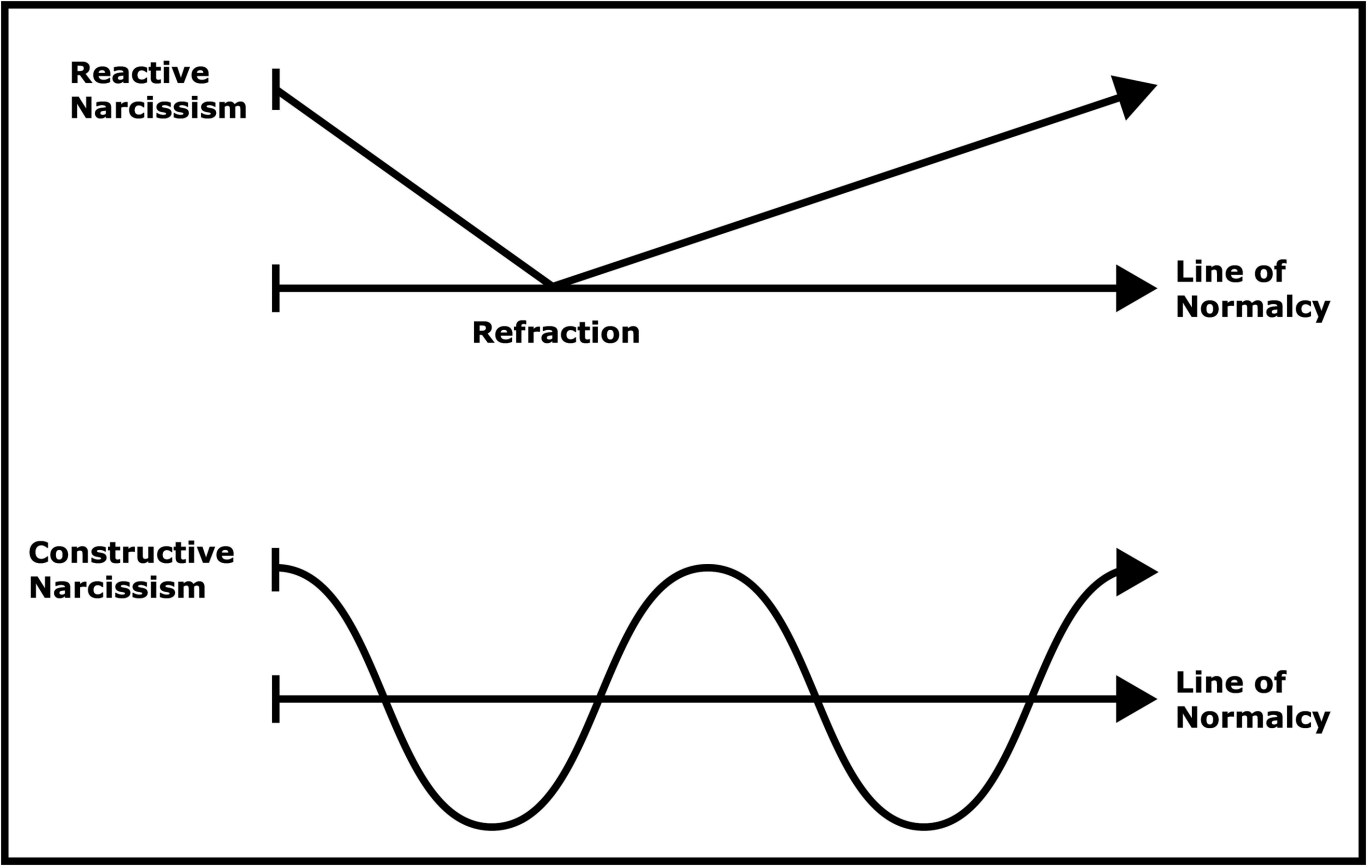
Understanding reactive and constructive narcissism.

Human beings live in a state of balance, both physically and psychologically. Just as a person with very high blood pressure is considered to be ill, a mental imbalance also indicates that a person is sick. In the psychological literature, this state of balance is referred to by many people (Lacan, 2007; Gruen, 2015; Fromm, 2017). We cannot say that all narcissism makes people sick. Here, we need to look at the types of narcissism. We can use Figure 1 to understand the types of narcissism and the characteristics of healthy and unhealthy narcissism:

As Figure 1 shows, we can analyze narcissism under two different headings: reactive and constructive. In Figure 1, we see a reactive narcissism that intersects the line of normality once and then moves further away from it with each passing day. On the contrary, in constructive narcissism, even though a person occasionally shows narcissistic characteristics, he/she constantly intersects with the line of normality and continues on his/her way in parallel with the line of normality. As shown in Figure 1, in reactive narcissism, a person moves further away from the line of normality over time due to a refraction experienced in childhood. In constructive narcissism, children who complete the narcissistic development phase in a healthy way continue their lives in parallel with the line of normality on the axis of self-esteem.

Narcissism is like a double-edged sword. Because the first periods of the human baby’s life are the periods of narcissistic development. It is vital to realize that a healthy dose of narcissism is essential for human development and helps to form the basis of self-esteem and personal identity. Too high or too low narcissism can destabilize a person’s balance, and when the balance is lost, instability regarding the sense of self at the core of the individual’s personality can develop (Carlock and Kets de Vries, 2007).

The point indicated as the refraction in Figure 1 shows the time when the dosage of narcissism starts to increase. On the other hand, the development close to the line of normality shown as constructive narcissism in Figure 1 represents healthy narcissism. Positioning narcissism directly in a “bad” place may make it hard for us to understand narcissism. Because a certain degree of narcissism, ranging from healthy self-esteem to destructive egoism, is entirely natural and even healthy. A balanced self-esteem contributes to positive behavior. In contrast to reactive narcissism, constructive narcissism develops as a result of sufficiently good care (Kets de Vries, 2006). Narcissism is not always pathological. Moreover, healthy narcissism produces behaviors such as humor and creativity (Rosenthal and Pittinsky, 2006). It can be stated that constructive narcissism in Figure 1 corresponds to healthy narcissism, and reactive narcissism corresponds to pathological narcissism.

Constructive narcissism develops in people who are sufficiently well cared for and experience age-appropriate frustration (Carlock and Kets de Vries, 2007). Reactive or pathological narcissism develops in people who have been psychologically damaged in some way. The parents’ unbalanced behavior, which can be called excessive, causes the child to create a defective sense of identity, and the child finds it difficult to maintain a stable sense of self-esteem. As they become adults, these children continue to be deeply disturbed by feelings of inadequacy, pain, anger, depressive thoughts, persistent feelings of emptiness and deprivation, and may develop an exaggerated sense of self-importance and self-importance to overcome these feelings (Kets de Vries, 2006). The clinical definition of pathological narcissism is based on a basic dysfunction related to intense approval and recognition (Wright et al., 2013).

Everyone has more or less narcissism (Hatemi and Fazekas, 2018). However, the narcissism referred to here is a self-esteem that does not harm others. In narcissism, which is called pathological or reactive narcissism, the person engages in a behavior that may cause problems for both himself/herself and others. We can use the Narcissistic Personality Inventory to measure the dosage of narcissism and to find out whether it is self-esteem or reactive narcissism. According to this inventory, if a person has characteristics such as grandiosity, authority seeking, love of authority, exhibitionism, exploitation, self-sufficiency, lack of empathy, feeling unique, having rights in everything, superiority, and arrogance, we can conclude that the person is a reactive narcissist (Raskin and Hall, 1979).

As can be understood from the Narcissistic Personality Inventory, narcissists are gods who worship themselves. It is already known that narcissism is called “God complex” (Levy et al., 2011). Narcissistic personality disorder is considered by psychotherapists to be the most difficult mental disorder to treat (McFarren, 1998). Narcissists cannot be cured because “Gods do not get sick.” Narcissism is considered to be the most difficult disease to treat because the causes of narcissism are hidden in the family or the childhood of the human infant.

## 3 What are the family causes of narcissism?

There is no one right way to understand narcissism; rather, there are various approaches that are more or less advantageous depending on their use (Hatemi and Fazekas, 2018). However, a general review of the literature shows that there is a close relationship between narcissism and family. The development of narcissism, especially in boys, is based on a particular family structure. Children with narcissistic development are extraordinarily dependent on the praise and attention of others to give them a sense of self because they have not sufficiently internalized a coherent sense of self or identity as children. Therefore, the relationships of narcissistic people are often exploitative and/or parasitic (Philipson, 1985).

In narcissistic development, the relationship between the child and the mother should be mentioned first. If the relationship between mother and child is maintained sufficiently well, the child is freed from narcissistic development and develops a healthy sense of self-esteem (McFarren, 1998). In contrast, the opposite situation develops when the child is exposed to excessive emotionality. Over-indulgence or over-protection of the mother towards the child may lead the child to unhealthy narcissism (Bianchi, 2014). We could argue the same thing about the father. In short, parents’ inconsistent emotional investments in their children and often interact with them to meet their own needs creates a sense of “devaluation” in the child. In such a condition, the child retreats defensively and begins to construct a pathologically grandiose self-representation (Levy et al., 2011). The relationship between narcissism, children, and parents can also be evaluated in the framework of the concept of attachment. The quality of parental behaviors triggers secure or insecure attachment phenomena in the child. Sensitive parents help construct a “secure attachment” in the child (Ainsworth et al., 1978). Beyond secure and anxious-resilient attachment models, a third model is the anxious-avoidant attachment model. In this model, the individual has no assurance that they will be helped when they ask for care; on the contrary, they expect to be rejected. When such an individual tries to live his life without the love and support of others, he tries to be emotionally self-sufficient and may later show narcissistic traits (Bowlby, 1988).

The mother is the child’s first approval mechanism (Kohut, 2009). In childhood development, parents, especially the mother or primary caregiver, are the first means of socialization. Children primarily use their emotions through their mothers. Children discover the answers to the most important existential questions that will shape their entire lives through their mothers. Children learn from their mothers how much they are loved, how potentially lovable they are, or how independent they can be. Narcissistic development refers to a stage of infancy through which we all must pass and through which growing children derive pleasure from their own bodies and functions. This early period is an extremely sensitive time in children’s lives, and the treatment they receive during this period colors their view of the world throughout their entire adult life (Carlock and Kets de Vries, 2007).

Babies’ sleep is narcissistic (Jacobson, 1964). Babies can be characterized as “narcissistic” because they live purely need-orientated lives and because babies act in line with their physical needs. In other words, for babies at this stage, there is only themselves and their needs in the world. Over time, infants complete their narcissistic development and move into a healthy state of self-esteem. Many of the human emotional factors are rooted in the individual’s past, especially in childhood, and are processed unconsciously (Kakar, 1977). However, children who do not acquire healthy self-love never value themselves as themselves but only as an extension of their parents. Children who do not overcome the narcissistic developmental stages in a healthy way experience self-dissatisfaction in adulthood and become defensive grandiosity, and when their defenses fail, they become severely depressed, paranoid, and angry (Loewenstein, 1997).

**Figure 2:**
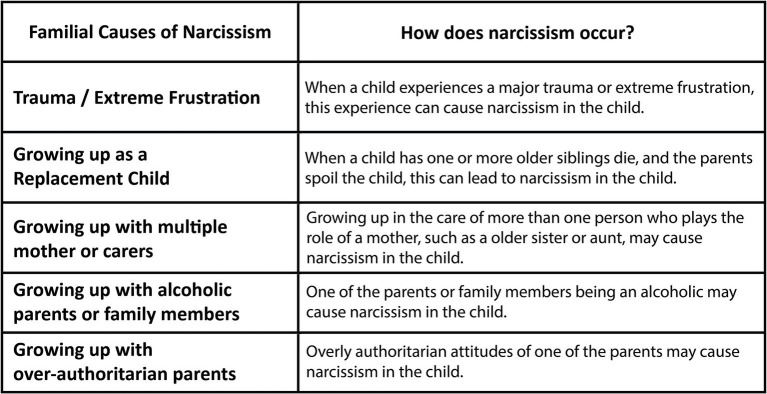
Family-related causes of narcissism.

Narcissistic development is a process that every person goes through. However, what is important is how this process is experienced. Since this process is generally spent within the family, what determines whether people will be narcissistic or not depends on the relationship they have with their parents and family members during childhood. Many things have been suggested as the cause of narcissism in the literature. However, in this study, I focus on familial causes of narcissism since I will compare political leaders in terms of their childhood, family, and narcissistic leadership. To better understand the familial causes of narcissism, we can make use of Figure 2.

As can be understood from Figure 2, we can list five different reasons among the familial causes of narcissism. We can explain these reasons as (1) experiencing trauma or having not age-appropriate frustrations, (2) growing up as a replacement child, (3) growing up with multiple mothers or carers, (4) growing up with alcoholic parents or family members, (5) spending childhood with overly authoritarian parents.

The first familial cause of narcissism is trauma or not having age-appropriate frustration in the child. An early trauma jeopardizes the formation of the nucleus of trust in the child, and a destructive and sadistic superego fills this deficiency in the psyche (De Cesarei, 2005). Children are unable to regulate their self-esteem due to early traumatic experiences (Levy et al., 2011). The traumatized person is excluded from society (Ferenczi, 2006). Trauma does not only occur in childhood. There is no age in trauma (Fromm, 1991). However, traumas experienced in childhood can affect people more.

Children who experience trauma or not age-appropriate frustration may experience refraction before completing their narcissistic development. Children acquire their sense of identity gradually and through interaction with the environment. To ensure normal character development, children need to resist challenging forces and experience both success and frustration. Moderate frustration, sometimes called age-appropriate frustration, is also necessary for mental health. It can be frustrating, for example, when a child has to wait a few minutes for his or her mother to finish something before putting lunch on the table. However, the level of frustration will not be inappropriate. But if the wait lasts for hours and the mother goes on a tirade about the disturbingness of small children, the frustration can be unhealthy and possibly even traumatizing (Kets de Vries, 2006).

The second of the familial causes of narcissism is that the child grows up as a replacement child. A mother who has already witnessed the death of several children may treat her newborn child as a love object (Loewenstein, 1997). In this situation, which is defined as “replacement child syndrome,” parents treat their newborn child as “God” because they have lost several children before, and the child is exposed to excessive emotionality. Children who grow up as substitute children may show narcissistic characteristics in adulthood because they cannot overcome their narcissistic development in childhood in a healthy way (Volkan, 2020).

The third familial cause of narcissism is that children spend their childhood with more than one mother or carer. This so-called “multiple mother syndrome” (Volkan, 2020) disrupts the emotional balance between children and their carers and causes children to be exposed to an unbalanced emotional state. When we consider that the role of parents and carers is very critical during narcissistic development, we can assume that children who grow up with multiple mothers are exposed to an unbalanced emotional state. Whether parents or carers are supportive and consistent or rejecting and inconsistent affects children’s narcissistic development. Family circumstances can expose children to traumatic experiences (Carlock and Kets de Vries, 2007). Hence, it can be stated that children who grow up with more than one mother may witness unbalanced emotional states and, therefore, may have unhealthy narcissistic characteristics. Two of the familial causes of narcissism are inversely related to each other. “Growing up with more than one mother or carer” and “growing up with an overly authoritarian parent” cannot occur at the same time. This is because children who grow up with an overly authoritarian father cannot be expected to have more than one carer.

The fourth familial cause of narcissism is that one of the parents is an alcoholic. This so-called “alcoholic parent syndrome” (Houghton, 2014) may negatively affect the narcissistic development of children. An alcoholic parent is unable to provide adequate and good care for the children. Children who do not get good enough care and experience age-inappropriate frustrations may have narcissistic traits (Carlock and Kets de Vries, 2007). This syndrome, referred to in the literature as “alcoholic parent syndrome,” is assumed to occur only if the mother or father is an alcoholic. However, in this study, we can claim that the fact that one or more of the older sisters or brothers of the children were alcoholics also negatively affected their narcissistic development. Because if someone in the family is an alcoholic, it can disrupt the normal emotional atmosphere in the family.

The fifth and last of the familial causes of narcissism is the growth of children in the hands of over-authoritarian parents. According to this condition, defined as “over-authoritarian parent syndrome,” (Gruen, 2014), children growing up with an over-authoritarian parent may cause narcissism in children. In the opposite cases, narcissism is also encountered. Children exposed to inadequate or uncaring parenting may later come to believe that they cannot count on anyone’s love or loyalty and act on this belief as adults. Children who live this way, although they claim to be self-sufficient, are disturbed by a sense of deprivation, anger, and emptiness in the depths of their being. They focus on power, beauty, status, prestige, and superiority to cope with these feelings. They may conceive of themselves as grandiose, exaggerate their achievements, need excessive admiration, and have an unrealistic sense of entitlement (Carlock and Kets de Vries, 2007).

Obviously, there is no such thing as a perfect parent, and becoming an individual is different from the cozy period of intrauterine existence when the child’s every need is met. The child’s growth does not occur without a certain amount of frustration, and normal development requires tolerable doses of frustration (Carlock and Kets de Vries, 2007). However, children who grow up with an over-authoritarian parent, especially those who experience violence in the family, are closer to narcissism. We know that children who have been victims of violence in childhood begin to seek power as adults and seek to prove themselves to rebuild their self-esteem, which their parent’s behavior has severely damaged (Kakar, 1977). Surprisingly, both children who are victims of physical violence and children who are spoilt can have narcissistic traits (Kets de Vries, 2006). From this point of view, we need to underline again the concept of emotional balance as an antidote to narcissism.

## 4 How did their parents impact the narcissistic political leadership of Hitler, Putin, and Trump?

Like all of us, political leaders occupy a position somewhere on the narcissistic spectrum, ranging from healthy self-confidence to pathological egoism (Kets de Vries, 2006). However, the boundary between political leadership and narcissism is not very clear. Because if political power and the power it gives to people become uncontrolled, it is improbable that the owner of that power will not show narcissistic characteristics. Power awakens the narcissistic parts of the human being and even creates addiction (Wardetzki, 2017). Probably for this reason, in studies analyzing the relationship between narcissism and political leadership, it is claimed that most of the heads of state in the 20th and 21st centuries were narcissists. In addition, the hubris syndrome, also known as a leader’s disease, should also be mentioned. In this context, it is worth noting that the relevant qualities of hubris and narcissism overlap. It can be stated that narcissism contributes to the development of hubris (Picone et al., 2014). Moreover, hubris and narcissism can coexist, but the precise nature of their relationship or co-occurrence has not yet been established. However, vital evidence points to the co-occurrence of hubristic and narcissistic leadership (Sadler-Smith, 2019).

**Figure 3:**
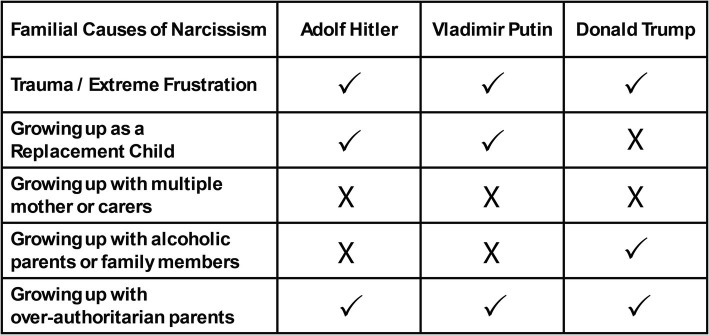
Familial causes of the narcissistic leaderships of Hitler, Putin, and Trump.

In addition to political leaders, it is also stated that many leaders of terrorist organizations are narcissists. In the context of terrorist organization leaders and narcissism, discussions about Osama bin Laden are prominent (Miliora, 2004, p. 125; Langman, 2021, p. 3). The concept of uncontrolled power can explain the fact that the leaders of almost all terrorist organizations are narcissists. However, we can also state that legitimately elected political leaders have not been able to move away from narcissism. Whether the person we are talking about is the leader of a terrorist organization or a legitimate political leader, we must remember: “They were once children, too.” It is possible to predict the policies to be implemented by a leader by looking at the leader’s family. Of course, our intention here is not to legitimize the actions of terrorist organization leaders or Hitler. Our primary purpose is to make a prediction about the future. To make this prediction, we need to understand to what extent the narcissistic political leadership of Hitler, Putin, and Trump was affected by their parents and families. We can use Figure 3 to explore the familial and parental reasons for the narcissistic leadership and politics of Hitler, Putin, and Trump:

As can be seen from Figure 3, it is pretty simple to compare Hitler, Putin, and Trump on the axis of familial causes of narcissism. Because, like Hitler, Putin, and Trump’s narcissistic characters and political leaderships depend on their families and parents. When we look at the childhoods of these three leaders in the axis of trauma and age-inappropriate frustration as a cause of narcissism, we can state that all three leaders were traumatized and experienced age-inappropriate frustrations.

We know that Hitler was an abused child, that his father physically abused Hitler Daily (Dörner and Güss, 2011), and that Hitler’s father beat Hitler with a belt made of hippopotamus skin (Gruen, 2022). As a result of his traumatization, Hitler had “chronic narcissistic rage” (Dreijmanis, 2005). It is possible to argue similar theses for Putin as for Hitler. Putin tells how his father beat him with a belt when he was a preschooler (Volkan, 2020). We can claim the same theses for Trump as for Hitler and Putin. “Trump often suggests violence in his rallies and in his tweets, and while there are constraining factors both inside and outside of the rallies themselves, the potential for violence is plain” (Goethals, 2021, p. 242). Although there is no indication that Trump’s father physically abused him, we know that he was put in a boarding school during his adolescence. The trauma caused by Trump being given to boarding school at an early age is interpreted as follows:

The cartoonists who drew Trump as a wounded little boy who wanted to be the most powerful man in the universe as compensation understood him well. At 12, Trump was expelled by his father from the luxurious family home and had to attend a military boarding school known for its strict discipline. It meant falling out of favor, being suddenly expelled from heaven while his brothers and sisters continued to enjoy the family luxuries. Trump will have wanted to get his rematch by being more successful than his father (Hirigoyen, 2019).

Hirigoyen wrote the above statements to explain the reasons for Trump’s narcissistic character and political leadership. However, we can remove Trump’s name from the above paragraph and write Hitler’s or Putin’s name. Because both Putin and Hitler, like Trump, can be described as little children who want to prove to their fathers that they are strong.

When Hitler, Putin, and Trump are compared under the heading of “growing up as a replacement child,” which is the second familial cause of narcissism, it is seen that Hitler and Putin are similar. We know that parents who have already had one or more children die put their next child in the place of “God,” thus exposing them to a disproportionate amount of emotionality, and that the child develops a “God complex” later in life. Hitler was born after his mother had lost her first three children (Macleod, 2005). Therefore, child Hitler became the sole object of love for his mother, Klara Hitler. His mother, Klara Hitler’s fondness for Adolf is generally recognized by historians (Loewenstein, 1997). Similar to Hitler, Putin grew up as a replacement child. Putin’s parents lost two children before his birth. The first, Albert, died as an infant before the Second World War; the other, Viktor, died in the blockade of Leningrad. Putin was born in 1952 when his mother was 41 years old and grew up listening to his parents’ bitter conversations about what had happened to the family during the war (Volkan, 2020). We know that Trump is not a replacement child. However, we know that Trump’s older brother, Fred Trump, passed away at a young age.

We do not have much data on the third familial cause of narcissism, “growing up with multiple mothers,” and the fourth familial cause of narcissism, “growing up with an alcoholic parent or family member.” We can say that it is not possible for these three leaders, who spent their childhoods with overly authoritarian fathers, to have more than one carer in their lives. However, it is possible that Putin’s and Hitler’s fathers were so violent that they beat them with a belt because of alcohol. We also know that Trump’s older brother, Fred Trump, who died at a young age, died of alcoholism.

When Hitler, Putin, and Trump are compared under the heading of “growing up with an authoritarian parent,” which is one of the most prominent causes of narcissism, a similar family structure is encountered. The fathers of these three leaders are extremely authoritarian, while their mothers paint a more compassionate and moderate portrait (Loewenstein, 1997). “I did not like my father very much, but I feared him. He threw tantrums and was physically violent” (Dreijmanis, 2005). “My mum’s death was a terrible blow to me” (Macleod, 2005). Hitler, Putin, or Trump may have made these statements. However, these statements came from the mouth of Hitler.

The political leadership of children who do not grow up in a balanced emotional state has always been problematic and will continue to be. In particular, people who experienced violence in their childhood with over-authoritarian parents attempt to prove themselves in parallel with the “Narcissistic Personality Inventory” (Raskin and Hall, 1979) when they grow up. When such persons become political leaders, they assume the role of savior or messiah. Hitler’s risky decisions and his need to prove himself are due to his failure to complete his narcissistic development in childhood healthily (Dörner and Güss, 2011). We can make the same observation for Trump (Williams et al., 2020). Trump makes very offensive comments during election campaigns (Hart and Stekler, 2021). However, the reason for Trump’s arrogant and aggressive behavior is his failure to complete his narcissistic development healthily.

Hitler knew everything. Because he had the desire for a messiah (Miliora, 2004). Hitler never admitted his wrongdoings. He even wrote in his suicide note, “My generals betrayed me” (Dörner and Güss, 2011). Similarly, Trump is omniscient and is seen by his followers as a “savior” (Maratea, 2024). Trump’s obvious lies and exaggerations, his own narcissism and self-aggrandizement, his putdowns of women or the disabled, are overlooked or even celebrated (Goethals, 2017, p. 6). However, Trump’s efforts to prove himself have not escaped the attention of a group of psychiatrists in the United States. In 2017, as soon as Donald Trump assumed the presidency of the United States, a group of eminent psychiatrists in the United States warned of the social, cultural, and geopolitical dangers of a psychologically abnormal US president. Years later, these warnings proved to be prescient (Lee, 2023).

Considering the damage caused by Hitler to world politics, the warnings made for Trump should be heeded. There are many warnings that Trump’s political leadership has fascist characteristics and will be problematic in the future (Tourish, 2024, pp. 20–24). Similar warnings have been made about Putin, similar to Trump. Because the narcissism of Putin’s political leadership is also evident. To understand the enigmatic politics in the Kremlin, we must first understand the extraordinary tension in Putin’s psychological structure. Putin has a fundamental insecurity, an inferiority complex, and a need to dominate and control. Putin’s political style is the direct result of his uncanny psycho-logical chemistry (Laqueur, 2015). In other words, Putin’s leadership is also a narcissistic political leadership, and it cannot be evaluated independently of his parents.

The narcissistic political leadership of Hitler, Putin, and Trump has almost common familial origins. Hitler’s narcissism led to the deaths of millions of people. Although Hitler’s genocide of the Jews has been attributed to his mother’s doctor or his falling in love with a Jewish girl, we know that these are not true (Macleod, 2005). Hitler inherited his inner violence from his family. However, we cannot claim that almost everyone who grows up in a family similar to Hitler’s family will be like Hitler. In addition, we must repeatedly emphasize that the similarities between the family structures of these three leaders and the attitudes of their parents cannot be ignored.

When a leader’s extreme narcissistic tendency is combined with their position of power, destructive consequences are inevitable (Kets de Vries, 2006). Even an ordinary public employee is subjected to 40 different health tests in all countries. Indeed, the intervention of psychiatry in politics has caused significant problems in China, Soviet Russia, and the USA, leading to the inhumane treatment of dissidents and blacks during slavery (Andrade and Campo-Redondo, 2020). However, it is still a necessity that there are institutions that offer a narcissism measurement on the axis of leader candidates, their childhood periods, their families, and their relations with their parents and that this information is openly shared with the public.

## 5 Conclusion

Not all narcissisms are bad. Because all children go through a stage of narcissistic development, some children manage to complete this phase healthily and proceed with their lives with healthy narcissism. Others are infected with reactive or pathological narcissism as a result of the refractions they experience in their narcissistic developmental stages. Healthy narcissism leads to self-esteem and creative character building. Unhealthy narcissism leads to grandiosity, the need to prove oneself to parents, exhibitionism, and a desire for power and authority.

If we liken a person to a building, we can say that the foundation of that building is buried where the family members and parents of that person are. This rule also applies to political leaders. The policies leaders implement and the kind of political leaders they become are determined by their childhood, their relationships with their families, and, in particular, the care they get from their parents. Children who have a traumatic past, who grow up as replacement children, who grow up with multiple mothers, who grow up with an alcoholic parent or family member, and most significantly, who grow up with an authoritarian parent are likely to become narcissistic political leaders if they become leaders. In other words, we can say that children who are deprived of adequate and balanced care during their childhood may become narcissistic political leaders if they become political leaders. We can list the similarities in the familial reasons for the narcissistic leadership of Hitler, Putin, and Trump as follows:

- Hitler, Putin, and Trump had traumatic childhoods.
- Hitler and Putin grew up as replacement children.
- Hitler and Putin were physically violated by their fathers.
- Donald Trump was sent to a military boarding school precisely during the period of narcissistic development. Therefore, we can say that Trump’s sense of secure attachment has been damaged.
- Fred Trump, Donald Trump’s older brother, died of alcoholism.
- Hitler, Putin, and Trump spent their childhood with an overly authoritarian father. For this reason, growing up with multiple mothers or carers, which is another familial cause of narcissism, was not the case for these three leaders.

Hitler bled the twentieth century as a narcissistic political leader because he lacked adequate/balanced care. Hitler’s actions can never be justified. However, it is impossible to fully understand Hitler’s actions without considering his relationship with his parents. Hitler’s narcissistic political leadership cannot be considered in isolation from the fact that he was a substitute child, that he was made a love object by his mother, and that he was constantly beaten by his father with a stallion-skin belt.

Similar to Hitler, Putin’s narcissistic political leadership cannot be analyzed apart from the fact that he was deprived of adequate/balanced care, was physically abused by his father as a preschooler, grew up as a replacement child, and was turned into a love object by his mother. Like Hitler and Putin, all indications point to the fact that Trump was deprived of adequate and balanced care as a child. Trump’s narcissistic political leadership cannot be viewed in isolation from the trauma of being sent to a boarding and disciplined school as an adolescent, the death of his older brother Fred Trump due to alcoholism, and his childhood with authoritarian parents.

Hitler’s narcissistic political leadership cost the lives of millions of people. While I was writing this article, many people died in Ukraine. Trump’s political rhetoric after his re-election clearly shows that many will suffer. We cannot prevent children who cannot complete their narcissistic development in a healthy way from becoming political leaders in their adulthood. However, we can underline once again that children like Hitler, Putin, and Trump, who are far from adequate/balanced care, may become narcissistic political leaders, and this may have many negative consequences both in the national and international political arena. Ultimately, it should not be forgotten that the similarities in the familial reasons for the narcissistic political leadership of these three figures could lead to policies similar to those implemented by Hitler. In this context, we can reiterate that the academic texts and warning statements written with concern by psychiatrists and psychiatric associations should be taken into account.
